# Serous cystadenocarcinoma of the pancreas: report of a case and management reflections

**DOI:** 10.1186/1477-7819-10-51

**Published:** 2012-03-08

**Authors:** K Bramis, A Petrou, A Papalambros, A Manzelli, E Mantonakis, N Brennan, E Felekouras

**Affiliations:** 1 LAIKON Hospital, First Department of Surgery, University of Athens Medical School, Greece; 2 HPB Surgery, Nicosia Surgical Department, Cyprus; 3 Hepatobiliary and Pancreatic Surgical Department, Oxford Radcliffe Hospitals NHS Foundation Trust; 4 Churchill Hospital, Oxford, UK

**Keywords:** pancreatic cancer, cystic tumors of the pancreas, serous cystadenoma, serous cystadenocarcinoma

## Abstract

**Background:**

Serous adenomas represent 1-2% of pancreatic neoplasms and typically are asymptomatic not requiring any treatment and simple observation is the option of choice. Although, they carry a realistic risk of malignancy despite the general view that they never become malignant. We report a case, which, according to our best knowledge is the 27th case reported in the literature.

**Methods:**

We reviewed the literature by performing a search in Pub Med and Medline.

**Results:**

A 86-year old patient known to have a serous cystadenoma of the pancreas treated conservatively through a close clinical and radiological follow up which was unattended for 4 years ending up to our emergency department suffering an acute abdomen. Exploratory laparotomy revealed a perforated prepyloric ulcer which was treated accordingly. Patient died some weeks later due to severe medical co morbidities.

**Conclusion:**

Serous cystic neoplasms of the pancreas carry a realistic risk of malignancy despite the general view that they never become malignant. In our opinion the treatment strategy of serous cystic neoplasms of the pancreas should be aggressive even in cases of remote metastases since prognosis of the disease is satisfactory

## Background

Malignant cystic tumors of the pancreas are very rare, accounting for about 1% of all pancreatic malignancies[1,2]. Pancreatic cystic neoplasms comprise 1-2% of pancreatic lesions [3] and most of these lesions are cystadenomas[3,4]. In 1978, Compagno and Oertel [5] issued an histopathologic classification of cystic neoplasms of the pancreas identifying two different types with different biological behavior. The first type is considered the serous cystic neoplasm and the serous cystadenoma represents numerically the most common entity. Also called glycogen-rich cystadenoma or microcystic adenoma is generally considered to be a benign condition although serous cystadenocarcinoma is a rare but known malignant condition described in the literature. Mucinous cystic neoplasms of the pancreas are the other type of lesions and possess the more frequent ability to transform to its malignant counterpart, namely mucinous cystadenocarcinoma [6]. Today serous and mucinous pancreatic neoplasms are classified according to the WHO nomenclature as tumors of the exocrine pancreas [7].

According to the latest WHO classification these tumors are called serous cystic neoplasms, which are cystic epithelial neoplasms composed of glycogen-rich, epithelial cells that produce a watery fluid similar to serum. Most of these are benign (serous cystadenomas), and in rare cases are malignant (serous cystadenocarcinoma). Serous adenoma has four variants: macrocystic serous cystic neoplasm, solid serous neoplasm, von Hippel-Lindau (VHL)-associated serous cystic neoplasm, and mixed serous-neuroendocrine neoplasm. The term serous cystadenoma refers to microcystic type (the most frequent) of the neoplasm [4].

In 1989, George et al [8] first reported a case of serous microcystic adenoma behaving in a malignant fashion and since then, according to our best knowledge, 26 cases have been reported in the literature. According to WHO the prevalence of malignancy of serous cystic neoplasms of the pancreas is 1%- 3% [4]. We report a case of serous cystadenocarcinoma with extensive local invasion and liver metastases.

## Case presentation

**Figure 1:**
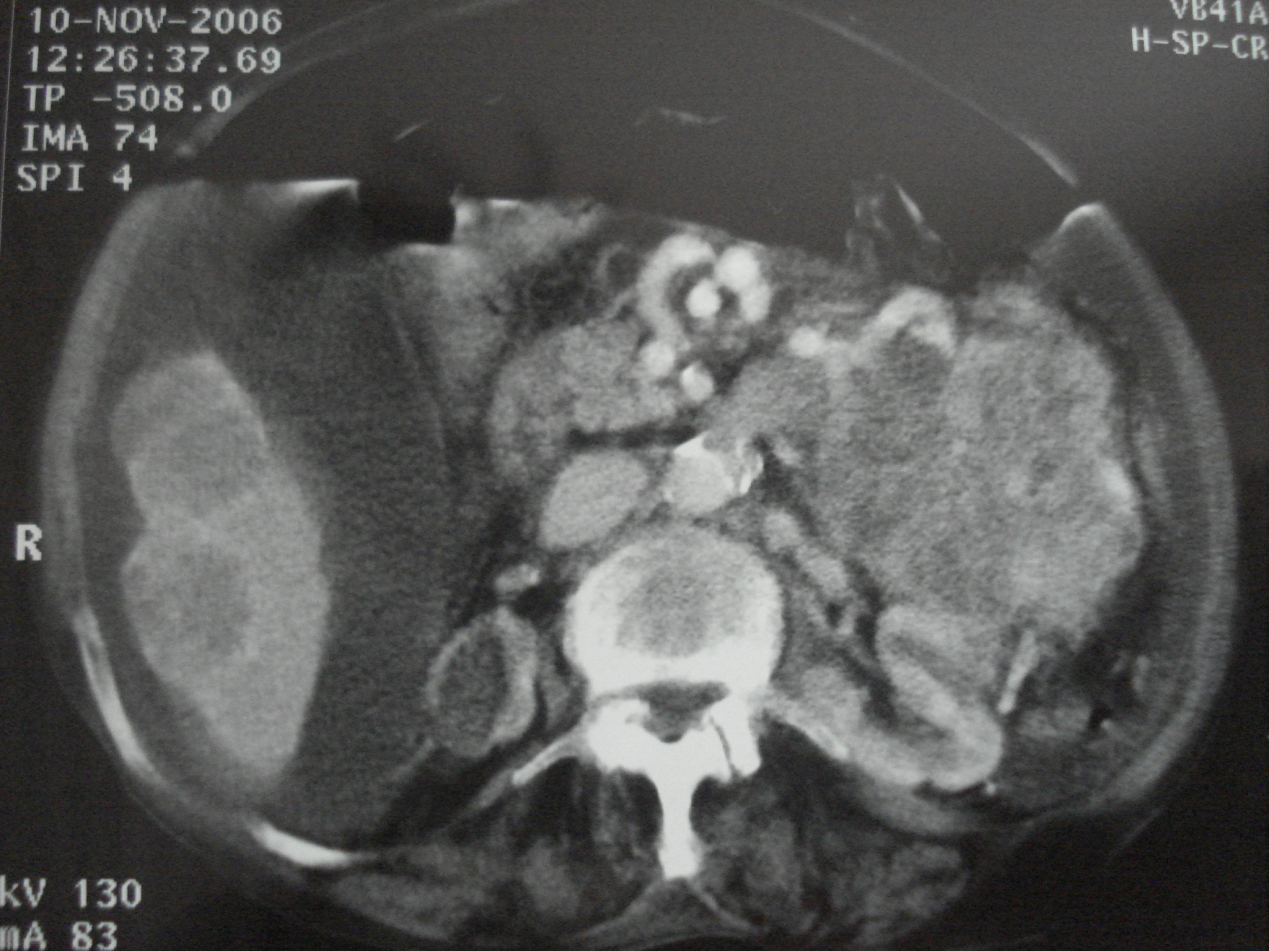
**Large amount of free peritoneal air and free fluid alongside with evidence o metastatic liver disease**.

**Figure 2:**
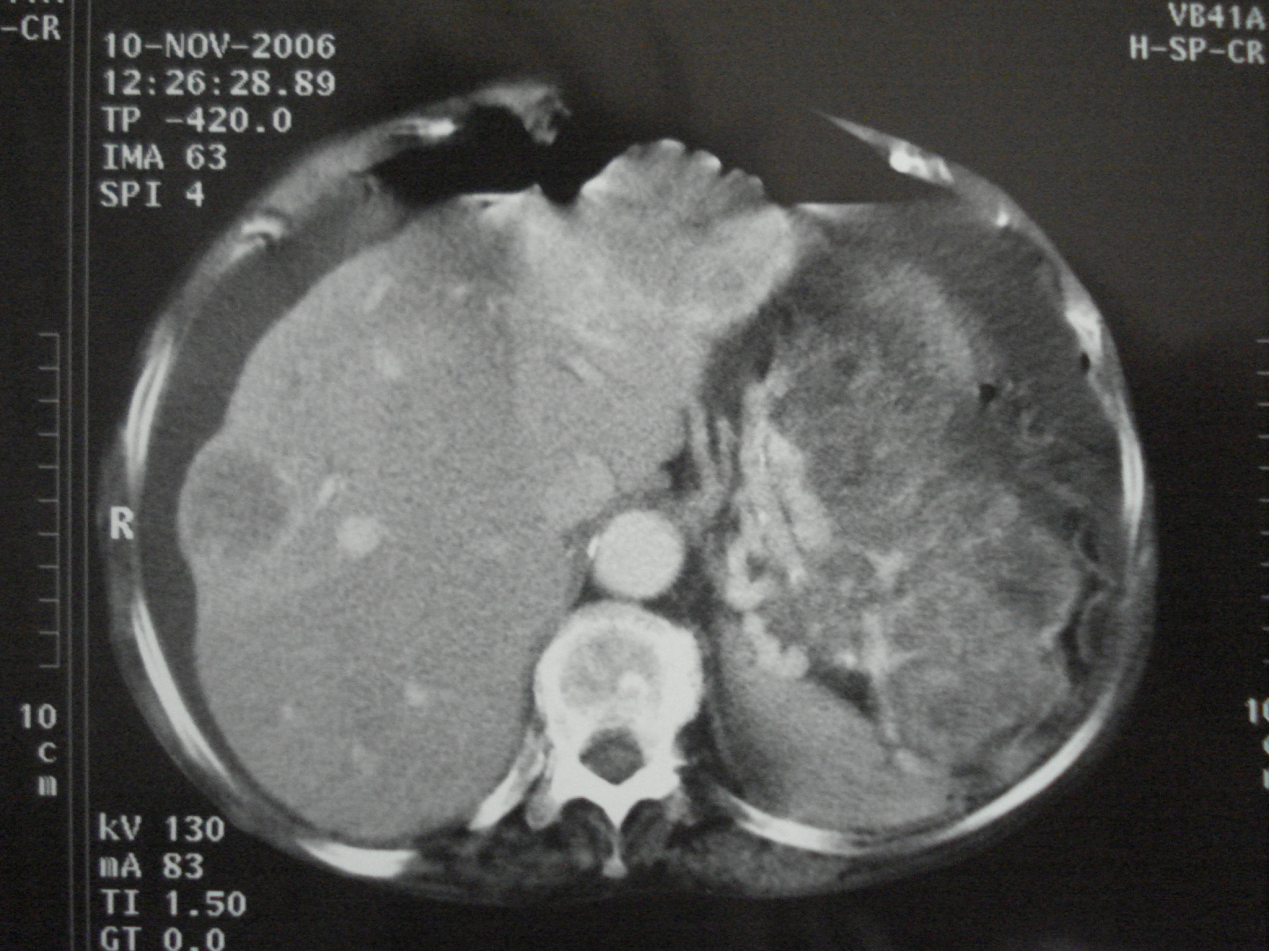
**A left upper quadrant 17 cm originating from the stomach along with multiple secondary liver lesions**.

An 86-year old woman presented at the emergency department suffering from severe acute upper abdominal pain. Her past medical history included hypertension, diabetes mellitus, coronary heart disease, atrial fibrillation, chronic obstructive pulmonary disease and twenty years before she had an omphalocele repair. The patient was in a close clinical and radiological follow up for monitoring a pancreatic serous cystadenoma diagnosed incidentally with a computed tomography (CT) scan 10 years earlier. The lesion was extensively studied with magnetic resonance imaging (MRI), endoscopic ultrasound (EUS), fine needle aspiration (FNA), serology and blood test screening. At that time she was addressed for a routine year follow up scan which she attended regularly for six years. Patient did not attend her follow up time table for the last four years. Upon admission to the emergency department, chest and abdominal x-rays were performed and turned out inconclusive. Routine laboratory studies revealed marked leukocytosis, severe anemia, and blood glucose levels exceeding 500 mg/dl. Due to inconclusive diagnosis, an intravenous contrast CT scan was ordered and revealed a large amount of free peritoneal air and free fluid. (Figure 1, 2) Moreover, a left upper quadrant 17 cm large mass originating from the stomach was revealed along with secondary liver lesions. An urgent laparotomy showed a prepyloric ulcer perforation and a large inoperable mass arising from the pancreatic body and largely invading the posterior wall of the stomach occupying the upper abdomen with multiple hepatic secondary lesions. A liver biopsy was taken, the stomach perforation was sutured closed and a washout was performed. The histopathologic findings from the biopsy material were consisted of small and medium size cystic lesions surrounded and divided by hyalloid collagenous matrix. The cysts were lined by cuboidal epithelial cells with clear cytoplasm and round nuclei without atypical or mitotic activity as demonstrated by pathology. The glycogen content was documented by PAS positive reaction. These findings were consistent with serous microcystic adenoma of the pancreas and the malignant nature of the tumor was confirmed by the presence of the extensive invasive large inoperable pancreatic mass with hepatic metastasis. The patient after a brief period in intensive care unit for respiratory problems recovered from surgery in 10 days but died a month later because of other, unrelated, serious medical problems.

## Conclusions

The prevalence of cancer among serous cystadenomas is reported to be 3% since the first case reported in the literature in 1989. It is rather clear from the literature that the differentiation between benign and malignant serous cystadenoma both histologically and clinically may be very difficult [9-11]. Additionally the presenting symptoms are not characteristic. To date serous cystadenoma is considered malignant when tumor invasion of surrounding tissues and organs or distant metastases are present [4]. According to the literature most cases of serous cystadenocarcinoma show synchronous or metachronous liver metastases, lymph node infiltration, splenic invasion, splenic vein infiltration and thrombosis, neural, perineural, and stromal invasion, as well as stomach, lung, adrenals, peritoneal, and colonic mesentery metastases [12-14]. Nowadays, the current management of serous cystadenomas of the pancreas is essentially conservative. In fact, once diagnosed correctly through CT scans, MRI, EUS, FNA, serology and blood test studies, observation and routinely reassessment of asymptomatic lesions is indicated thereby avoiding a major operation and resulting morbidity and mortality [1-3]. In our department we practice a six monthly follow-up. Nevertheless, clinicians should be aware of the possibility for malignant transformation in serous cystic neoplasms and should maintain a high index of suspicion when certain clues appear. These include the onset of new symptoms, worsening of symptoms, or rapid enlargement of the mass [15]. In these cases, resection may be indicated, despite the lack of objective evidence for malignancy obtained from preoperative imaging, endoscopy, and biopsies [3,13,15-17]. Our trust multidisciplinary specialist team board adopts and agrees with the conservative approach in the majority of the benign cases. Although, considering the chance of malignant transformation, our policy is to strictly discuss the treatment options with the patient and propose radical surgery especially in young and fit patients where the clinical and radiological situation of their lesions is minimally doubtful, unclear or borderline.

In the present case the course of the disease was indolent and only discovered after a co morbid condition requiring admission to the hospital. This case demonstrates that conservative management often does not have the success hoped for. In fact, the best results are guaranteed in those cases where patients are closely followed up and attended to their scheduled scans.

Cystic lesions of the pancreas are a heterogeneous group of disorders, newly defined, with varied biological behavior. The prevalence of such neoplasms is increasing. The reason is probably due to the spread and efficacy of diagnostic tools which frequently pick up, cystic pancreatic lesions in asymptomatic patients as incidental finding [17,18]. A thorough knowledge and experience on the matter by the surgical team is more than important in order to have a proper classification and treatment of these conditions. The morphological study of these tumors is based on the following investigations: ultrasound, CT scans MRI and EUS with FNA. Among this group of lesions, the serous cystadenoma is typically asymptomatic; not requiring any treatment and simple observation is the option of choice [19-21]. Serous cystic neoplasms of the pancreas although they carry a realistic risk of malignancy, the predominant view is that they never become malignant. In our opinion the treatment strategy of cystic serous neoplasms of the pancreas should be aggressive, although evidence for that is weak since it is largely based on case reports. Consequently, more studies are needed to set treatment guidelines for serous cystic neoplasms of the pancreas. Surgical treatment is considered necessary only for symptomatic patients or in cases of diagnostic doubt mainly because of the perioperative risks of radical pancreatic surgery especially when a benign disease is addressed. Whilst the management of intraductal papillary mucinous neoplasms and mucinous cystic neoplasms of the pancreas is regulated by an extended literature and even by international consensus guidelines, for the benign or borderline serous neoplasms of the pancreas a shared management vision is still needed. As mentioned, our clinical behavior has recently turned towards a greater focus on surgery. We consider that surgery of the body and especially the tail of the pancreas is technically safe and feasible with improved rates of morbidity and mortality and better impact on patient thanks to new surgical tools, better surgical materials, new technology and new advancements in surgery such as the minimally invasive access. Furthermore, pancreatic surgery is practiced in high volume centers with great experience, thus minimizing perioperative morbidity and mortality [22]. Thus, in selected patients with benign pancreatic serous cystic lesions of considerable size and a borderline clinical behavior, after a short period of clinical and radiological followup, we feel that a surgical treatment must be offered.

Considering the current numbers and the future trend of this matter, a consensus review of the management of the whole spectrum of the cystic pancreatic neoplasms is needed. Although, practice guidelines are missing, in cases of cystic serous cystadenomas, an extensive discussion with the patients on the management options must be offered.

## Consent

Written informed consent was obtained from the patient for publication of this case report and accompanying images. A copy of the written consent is available for review by the Editor-in-Chief of this journal.

## Competing interests

The authors declare that they have no competing interests.

## Authors' contributions

BK, PA, PA, FE were the surgical team involved in the management of the patient. ME performed the pathological assessment. BN and MA designed and co-wrote the paper. All authors read and approved the final manuscript.
